# Supplementary material for: Identifying Māori perspectives on gene editing in Aotearoa New Zealand
Source: Commun Biol. 2024 Feb 22;7:221. doi: 10.1038/s42003-024-05896-1 (PMC10883908; doi:10.1038/s42003-024-05896-1)
Supplement: Supplementary file 2 — Reporting Summary [file 42003_2024_5896_MOESM2_ESM.pdf]

nature portfolio

Corresponding author(s): Maui Hudson

Last updated by author(s): Jan 16, 2024

# Reporting Summary

Nature Portfolio wishes to improve the reproducibility of the work that we publish. This form provides structure for consistency and transparency in reporting. For further information on Nature Portfolio policies, see our Editorial Policies and the Editorial Policy Checklist.

## Statistics

For all statistical analyses, confirm that the following items are present in the figure legend, table legend, main text, or Methods section.

| n/a | Confirmed | |
|---|---|---|
| ☒ | ☐ | The exact sample size (*n*) for each experimental group/condition, given as a discrete number and unit of measurement |
| ☒ | ☐ | A statement on whether measurements were taken from distinct samples or whether the same sample was measured repeatedly |
| ☒ | ☐ | The statistical test(s) used AND whether they are one- or two-sided<br>*Only common tests should be described solely by name; describe more complex techniques in the Methods section.* |
| ☒ | ☐ | A description of all covariates tested |
| ☒ | ☐ | A description of any assumptions or corrections, such as tests of normality and adjustment for multiple comparisons |
| ☒ | ☐ | A full description of the statistical parameters including central tendency (e.g. means) or other basic estimates (e.g. regression coefficient) AND variation (e.g. standard deviation) or associated estimates of uncertainty (e.g. confidence intervals) |
| ☒ | ☐ | For null hypothesis testing, the test statistic (e.g. *F*, *t*, *r*) with confidence intervals, effect sizes, degrees of freedom and *P* value noted<br>*Give P values as exact values whenever suitable.* |
| ☒ | ☐ | For Bayesian analysis, information on the choice of priors and Markov chain Monte Carlo settings |
| ☒ | ☐ | For hierarchical and complex designs, identification of the appropriate level for tests and full reporting of outcomes |
| ☒ | ☐ | Estimates of effect sizes (e.g. Cohen's *d*, Pearson's *r*), indicating how they were calculated |

*Our web collection on statistics for biologists contains articles on many of the points above.*

## Software and code

Policy information about availability of computer code

| | |
|---|---|
| Data collection | Nil |
| Data analysis | Nil |

For manuscripts utilizing custom algorithms or software that are central to the research but not yet described in published literature, software must be made available to editors and reviewers. We strongly encourage code deposition in a community repository (e.g. GitHub). See the Nature Portfolio guidelines for submitting code & software for further information.

## Data

Policy information about availability of data

All manuscripts must include a data availability statement. This statement should provide the following information, where applicable:

- Accession codes, unique identifiers, or web links for publicly available datasets
- A description of any restrictions on data availability
- For clinical datasets or third party data, please ensure that the statement adheres to our policy

Survey data can be requested from the authors

## Research involving human participants, their data, or biological material

Policy information about studies with human participants or human data. See also policy information about sex, gender (identity/presentation), and sexual orientation and race, ethnicity and racism.

| | |
|---|---|
| Reporting on sex and gender | Survey data was reported on by gender in the original report but not within this commentary |
| Reporting on race, ethnicity, or other socially relevant groupings | The manuscript describes perspectives of Māori participants. Participants in all the qualitative activities were Māori and the survey was a mix of Māori and non-Māori participants. Information about the survey itself has been published. |
| Population characteristics | See above |
| Recruitment | For qualitative activities participants were either self-selected or invited via targeted recruitement or snowball techniques. |
| Ethics oversight | Ethical approval for the study was granted through Te Manu Taiko Ethics Committee at the University of Waikato |

Note that full information on the approval of the study protocol must also be provided in the manuscript.

# Field-specific reporting

Please select the one below that is the best fit for your research. If you are not sure, read the appropriate sections before making your selection.

☐ Life sciences ☒ Behavioural & social sciences ☐ Ecological, evolutionary & environmental sciences

For a reference copy of the document with all sections, see nature.com/documents/nr-reporting-summary-flat.pdf

# Behavioural & social sciences study design

All studies must disclose on these points even when the disclosure is negative.

| | |
|---|---|
| Study description | The manuscript describes a mixed method approach providing an analysis of a range of qualitative and quantitative research activities some of which have been previously published. |
| Research sample | This article summarises a range of research activities that had samples ranging from 10 to 830 as relevant to the specific activity. |
| Sampling strategy | A range of sampling strategies were used for different research activities. Targeted sampling of key informants, self-selection for SING participants, snowball sampling for various workshops, and randomised sampling for the survey |
| Data collection | The project began with a scoping exercise, involving a literature review and pilot interview-based survey, followed by a Summer Internship for Indigenous Peoples in Genomics (SING), and interviews with professionals at the interface of Te Ao Māori (the Māori world) and science, to identify the implications of genomic research for Māori communities (2019-2020). Alongside the interviews, the research team carried out a national survey of 830 Māori and non-Māori respondents to analyse 'how the diversity of values of a wide range of stakeholders on gene editing technologies [can] be negotiated to shape a robust policy design on the use and regulation of these technologies in a variety of sectors and contexts'.<br>The cultural aspect of the research was reinforced by: active engagement with Indigenous perspectives on the intersections of science, business, and Indigenous knowledge systems at a Māori thought-leaders workshop on genomics, taonga species and emerging commercialisation prospects (September 2019); an interactive session on gene editing at an Indigenous Genomics Conference (January 2020); a cultural wānanga ('sense-making workshop') in which cultural experts and community members considered how learnings from the research might be utilised in policy and practice (September 2020); and a primary sector hui (meeting, gathering) of representatives of Māori entities operating in the primary sector (forestry, horticulture, agriculture), to explore views on how gene editing and research could be commercially applied (December 2020). |
| Timing | Data collection activities occurred from Jan 2018 - Dec 2020. |
| Data exclusions | There were no data exclusions |
| Non-participation | Non-participation rates for the different data collection activities were not registered |
| Randomization | N/A |

# Reporting for specific materials, systems and methods

We require information from authors about some types of materials, experimental systems and methods used in many studies. Here, indicate whether each material, system or method listed is relevant to your study. If you are not sure if a list item applies to your research, read the appropriate section before selecting a response.

## Materials & experimental systems

| n/a | Involved in the study |
|---|---|
| ☒ | ☐ Antibodies |
| ☒ | ☐ Eukaryotic cell lines |
| ☒ | ☐ Palaeontology and archaeology |
| ☒ | ☐ Animals and other organisms |
| ☒ | ☐ Clinical data |
| ☒ | ☐ Dual use research of concern |
| ☒ | ☐ Plants |

## Methods

| n/a | Involved in the study |
|---|---|
| ☒ | ☐ ChIP-seq |
| ☒ | ☐ Flow cytometry |
| ☒ | ☐ MRI-based neuroimaging |

# Plants

| | |
|---|---|
| Seed stocks | N/A |
| Novel plant genotypes | N/A |
| Authentication | N/A |
